# Supplementary material for: The Immunoproteasome Is Expressed but Dispensable for a Leukemia Infected Cell Vaccine
Source: Vaccines (Basel). 2025 Aug 5;13(8):835. doi: 10.3390/vaccines13080835 (PMC12390170; doi:10.3390/vaccines13080835)
Supplement: Supplementary file 1 [file vaccines-13-00835-s001.zip › vaccines-3767824-Legends for Supplementary Figures (clean).pdf]

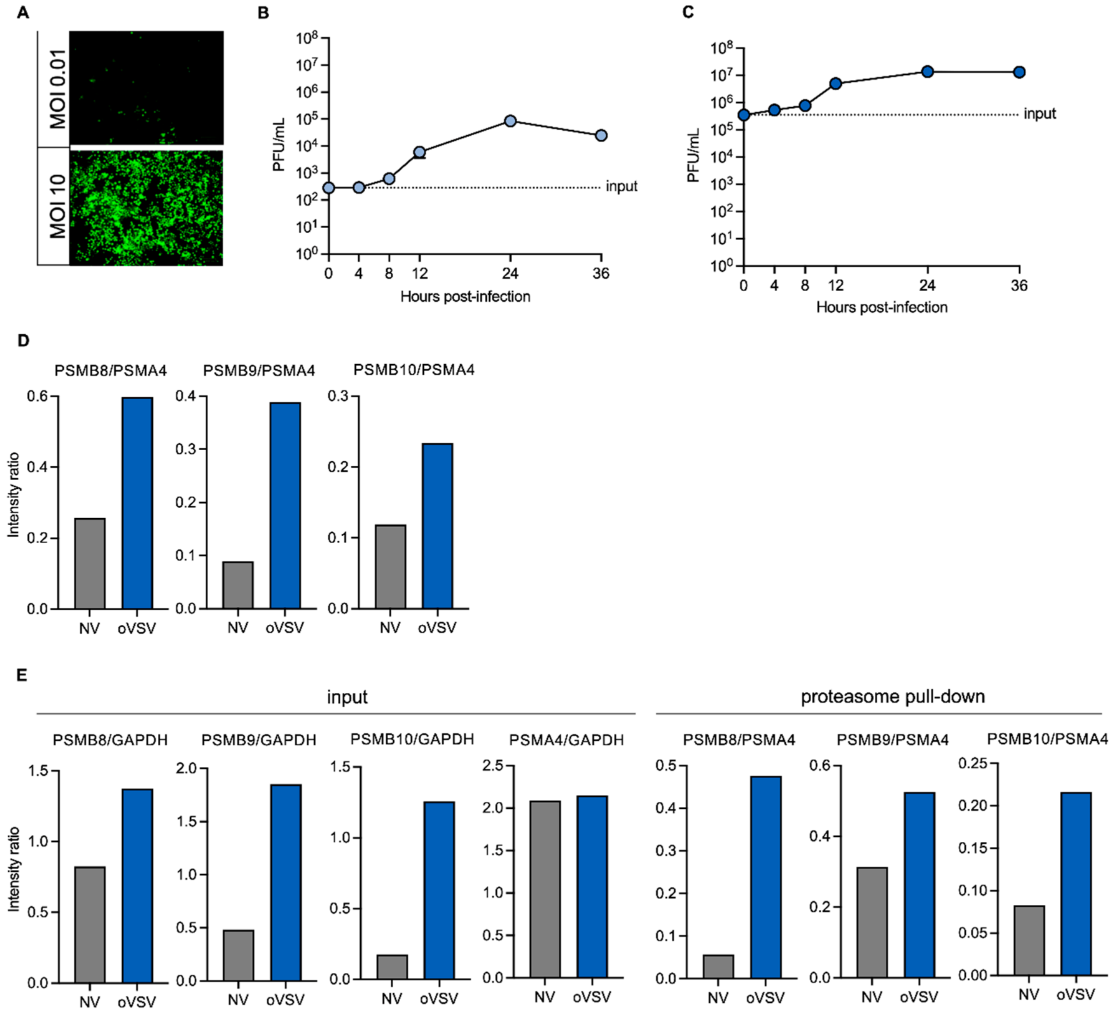

Figure S1: L1210 cells are sensitive to oVSV and IFN competent.

(A) Fluorescence images of L1210 cells infected with oVSV-YFP at MOIs of 0.01 or 10 for 24h. Images are representative of three independent infections. Virus growth curves of L1210 cells infected with oVSV-YFP at MOIs of (B) 0.01 or (C) 10 (n=3). Data are representative of two independent experiments. The dotted lines represent virus inputs. (D) Quantification of signal intensity for PSMB8, PSMB9 and PSMB10 from the Western blot shown in Figure 1B. Integrated density for each ImP subunit was normalized to the GAPDH protein loading control. (E) Quantification of signal intensity for PSMB8, PSMB9 and PSMB10 from the Western blot shown in Figure 1C. Integrated density for each ImP subunit was normalized to the PSMA4 proteasome loading control.

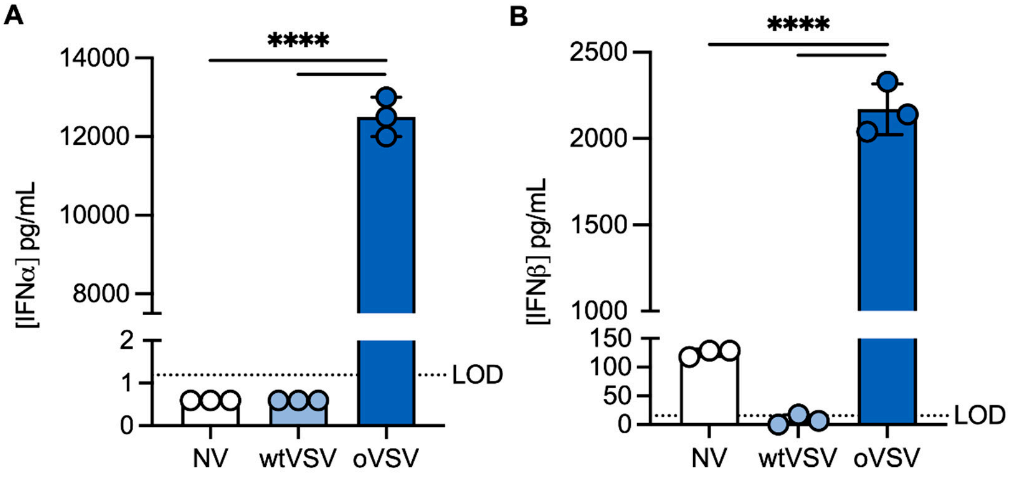

Figure S2: L1210 cells produce IFNα and β upon infection with oVSV but not wtVSV. A) IFNα or (B) IFNβ production by L1210 cells 24h post-infection with oVSV- or wtVSV-GFP at an MOI of 10 (n=3). Data are representative of two independent experiments. ****p<0.0001 (one-tailed unpaired t-test with Welch's correction). Non-statistically significant differences (p≥0.05) are not indicated on the graphs.

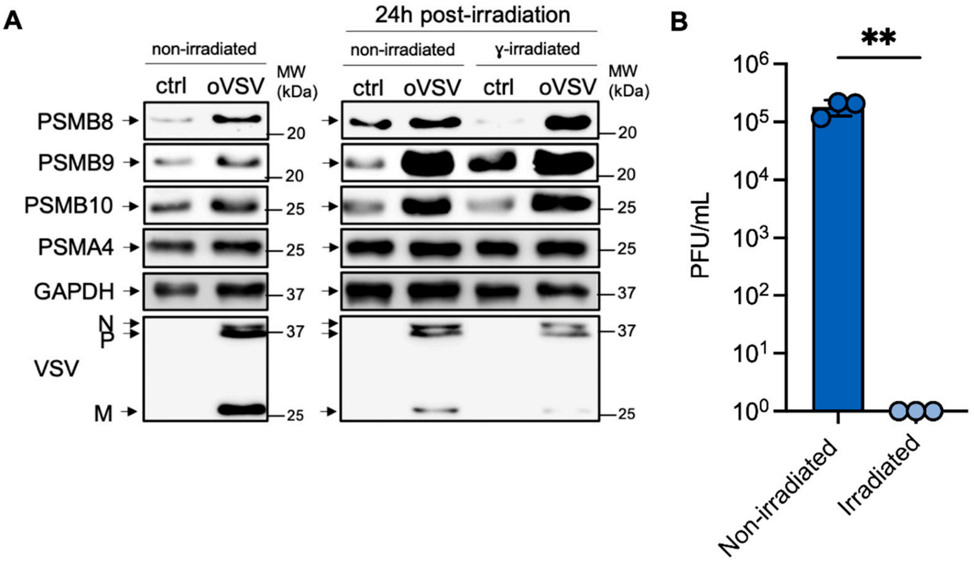

Figure S3: The ImP is induced in γ-irradiated cells but irradiated cells do not produce infectious virus. (A) Western blot analysis of PSMB8, 9 and 10 expression of L1210 cells

that were infected with oVSV-YFP at an MOI of 10 for 18h and then ɣ-irradiated and put back in culture for 24h. The left and right panels show ImP expression 18h post-infection and 24h post-irradiation (42h post-infection), respectively (representative of two independent experiments). (B) Virus production of L1210 cells from panel A at 24h post-ɣ-irradiation (n=3) (representative of two independent experiments). **p<0.01 (one-tailed unpaired t-test with Welch's correction). Non-statistically significant differences ($p \geq 0.05$) are not indicated on the graphs.

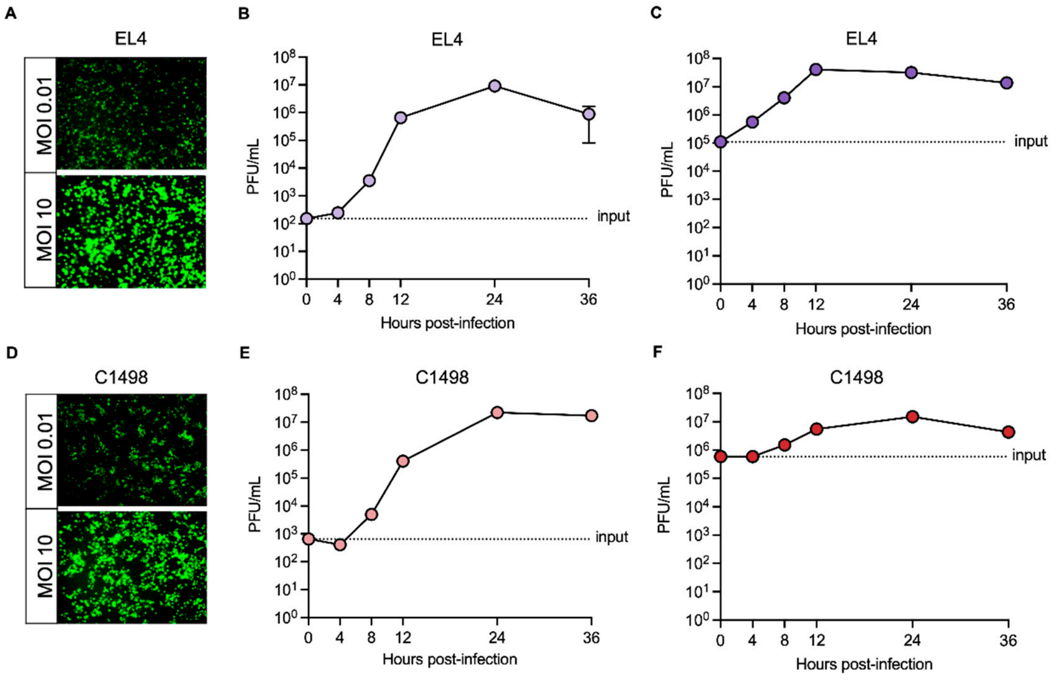

Figure S4: EL4 and C1498 cells are sensitive to oVSV-YFP infection.

(A) Fluorescence images of EL4 cells infected for 24h at MOIs of 0.01 or 10. Pictures are representative of three separate infections. (B) Viral growth curves of EL4 cells infected with oVSV-YFP at MOIs of 0.01 (n=3) or (C) 10 (n=3) (representative of two independent experiments). (D) Fluorescence images of C1498 cells infected for 24h at an MOI of 0.01. Pictures are representative of three separate infections. (E) Viral growth curves of C1498 cells infected with oVSV-YFP at MOIs of 0.01 (n=3) or (F) 10 (n=3) (representative of two independent experiments). The dotted lines represent virus inputs used for infection.

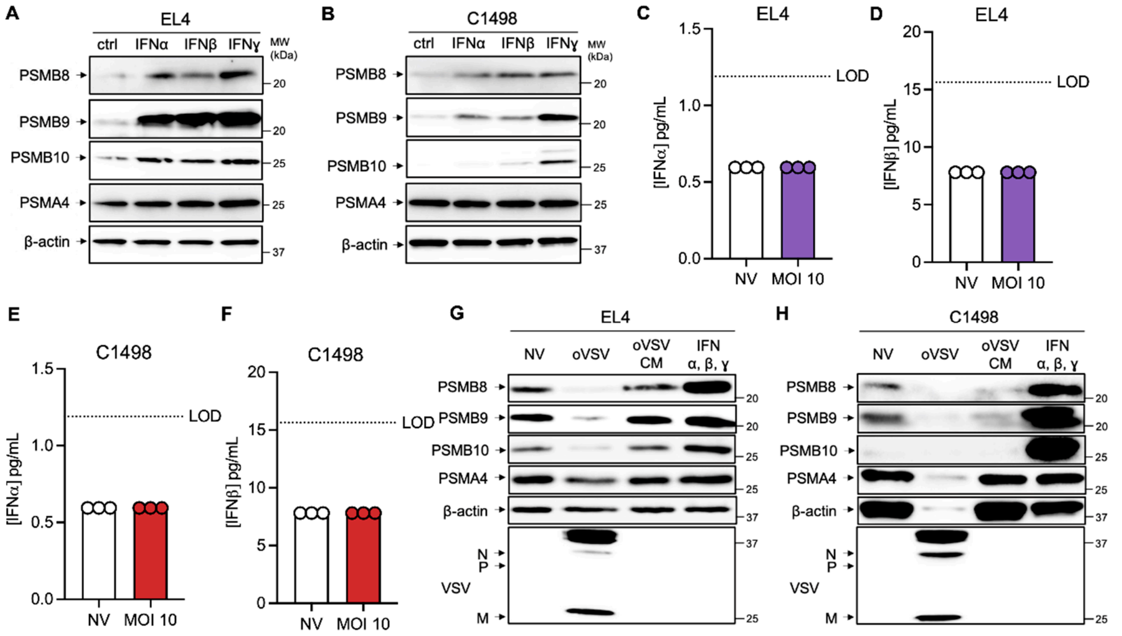

Figure S5: EL4 and C1498 cells are responsive to type I IFNs, but do not produce the cytokines post-oVSV-YFP infection. Western blot analysis of PSMB8, 9 and 10 expression by (A) EL4 and (B) C1498 cells 24h post-stimulation with IFNα, IFNβ or IFNɣ (representative of two independent experiments). (C) IFNα and (D) IFNβ production by EL4 cells 24h post-infection with oVSV-YFP at an MOI of 0.01 (n=3) (representative of two independent experiments) (E) IFNα and (D) IFNβ production by C1498 cells 24h post-infection with oVSV-YFP at an MOI of 0.01 (n=3) (representative of two independent experiments). Western blot analysis of PSMB8, 9 and 10 expression of (G) EL4 cells infected with oVSV-YFP at an MOI of 1 for 24h or stimulated with CM from oVSV-YFP-infected cells or stimulated with IFNs α, β and ɣ, or of (H) C1498 cells in the same conditions. Data are representative of two independent experiments. Statistical analyses were performed using the one-tailed unpaired t-test with Welch's correction for panels C, D, E and F but did not reveal significant differences ($p \geq 0.05$).

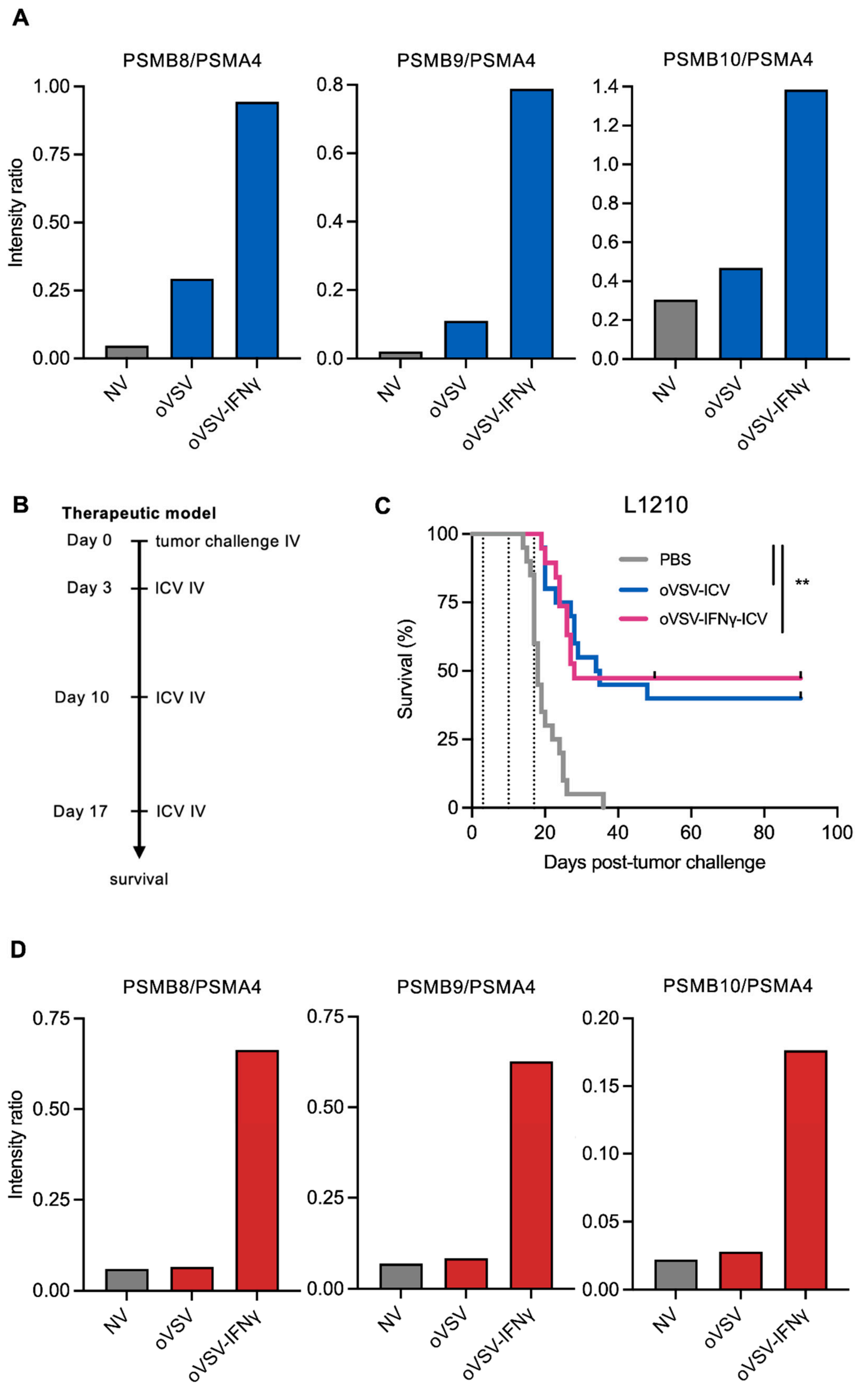

A
PSMB8/PSMA4
PSMB9/PSMA4
PSMB10/PSMA4
Intensity ratio
NV
oVSV
oVSV-IFNγ
B
Therapeutic model
Day 0
tumor challenge IV
Day 3
ICV IV
Day 10
ICV IV
Day 17
ICV IV
survival
C
L1210
Survival (%)
PBS
oVSV-ICV
oVSV-IFNγ-ICV
**
Days post-tumor challenge
D
PSMB8/PSMA4
PSMB9/PSMA4
PSMB10/PSMA4
Intensity ratio
NV
oVSV
oVSV-IFNγ

Figure S6: Therapeutic vaccination with oVSV-IFNɣ-ICV does not improve efficacy when initiated at day 3 post-challenge.

(A) Quantification of signal intensity for PSMB8, PSMB9 and PSMB10 from the Western blot shown in Figure 4A. Integrated density for each ImP subunit was normalized to the PSMA4 proteasome loading control. (B) Experimental timeline of the therapeutic vaccination regimen used in this figure. (C) Kaplan-Meier survival analysis of L1210-challenged mice treated with PBS (mock, n=20), oVSV-YFP-ICV (n=20) or oVSV-IFNɣ-ICV (n=19). The dotted lines indicate the days of vaccination. Data are representative of two independent experiments. **$p<0.01$ (log rank Mantel-Cox test). Non-statistically significant differences ($p\geq0.05$) are not indicated on the graphs. (D) Quantification of signal intensity for PSMB8, PSMB9 and PSMB10 from the Western blot shown in Figure 4D. Integrated density for each ImP subunit was normalized to the PSMA4 proteasome loading control.

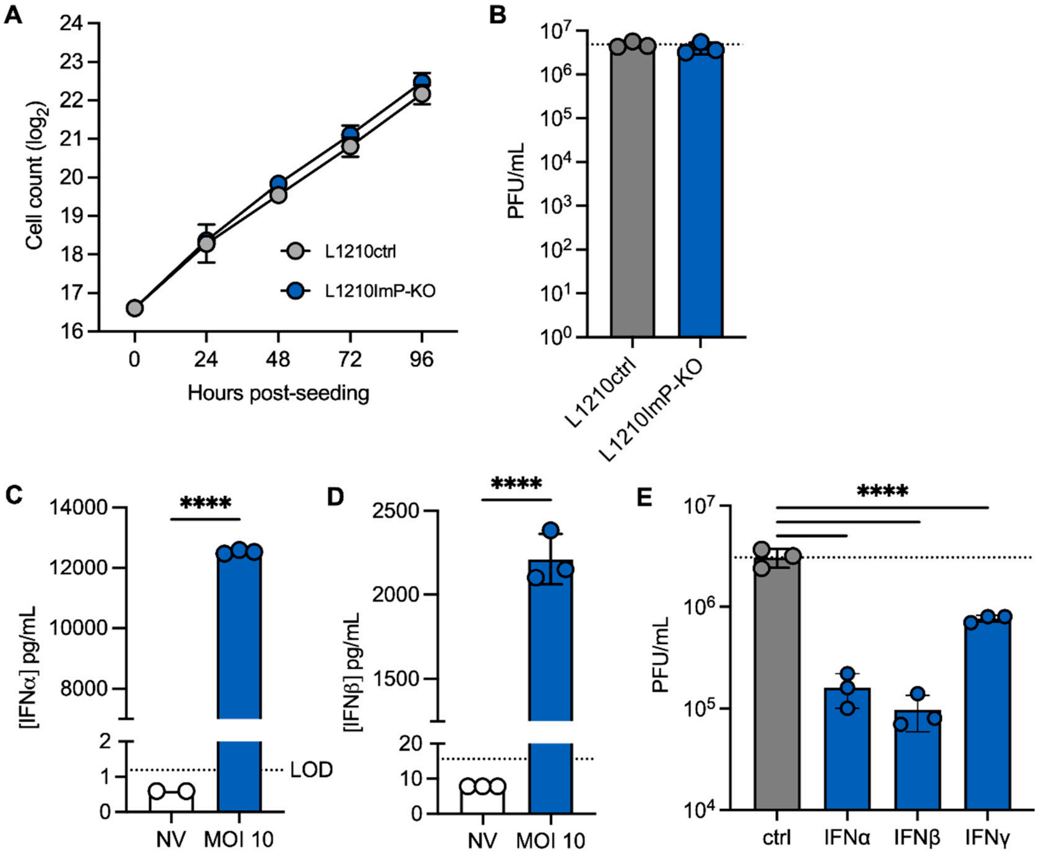

Figure S7: L1210ImP-KO and ctrl cells have similar growth rates, sensitivities to oVSV-YFP and are IFN competent. (A) Proliferation of L1210-ImP-KO and L1210ctrl cells (n=3). The dotted line indicates the number of cells seeded (representative of two independent experiments). (B) Virus production of L1210-ImP-KO and L1210ctrl cells at 24h post-infection with oVSV-YFP at an MOI of 10 (n=3). The dotted line indicates the average infectious virus production of the control cell line. Data are representative of two independent experiments. (C) IFNα or (D) IFNβ production by L210ImP-KO cells 24h post-infection with oVSV-YFP at an MOI of 10 as measured by ELISA (representative of two independent experiments). (E) Virus production by L1210ImP-KO cells pre-treated with IFNα, IFNβ or IFNγ and then infected with oVSV-YFP for 24h (n=3). The dotted line represents the average virus production by untreated cells. Data are representative of two independent experiments. Statistical analyses were performed using the 2-way ANOVA for (A) and the one-tailed unpaired t-test with Welch's correction for panels B, C, D and E (****$p<0.0001$). and $p \geq 0.05$ is considered non-significant and is not shown on the graph. Non-statistically significant differences ($p \geq 0.05$) are not indicated on the graphs.
